# mTORC2 protects the heart from high-fat diet-induced cardiomyopathy through mitochondrial fission in *Drosophila*

**DOI:** 10.3389/fcell.2022.866210

**Published:** 2022-07-15

**Authors:** Peiduo Liu, Kai Chang, Guillermo Requejo, Hua Bai

**Affiliations:** Department of Genetics, Development and Cell Biology, Iowa State University, Ames, IA, United States

**Keywords:** rictor, DRP1, Akt, mitochondrial dynamics, mitochondrial homeostasis, *Drosophila* cardiomyopathy, semi-automatic optical heartbeat analysis (SOHA)

## Abstract

High-fat diet (HFD)-induced obesity has become the major risk factor for the development of cardiovascular diseases, but the underlying mechanisms remain poorly understood. Here, we use *Drosophila* as a model to study the role of mTORC2 in HFD-induced mitochondrial fission and cardiac dysfunction. We find that knockdown of mTORC2 subunit *rictor* blocks HFD-induced mitochondrial fragmentation and Drp1 recruitment. Knockdown of *rictor* further impairs cardiac contractile function under HFD treatment. Surprisingly, knockdown of *Akt*, the major effector of mTORC2, did not affect HFD-induced mitochondrial fission. Similar to mTORC2 inhibition, knockdown of *Drp1* blocks HFD-induced mitochondrial fragmentation and induces contractile defects. Furthermore, overexpression of *Drp1* restored HFD-induced mitochondrial fragmentation in *rictor* knockdown flies. Thus, we uncover a novel function of mTORC2 in protecting the heart from HFD treatment through Drp1-dependent mitochondrial fission.

## Introduction

The World Health Organization (WHO) reported that around 32% of the total death is caused by cardiovascular diseases (CVDs), which makes CVDs the leading cause of death. Dietary conditions like a high-fat diet (HFD) can lead to various heart dysfunctions, including increased heart rate, high ejection fraction, cardiac hypertrophy, increased heart fibrosis, and elevated triglyceride content (Ouwens et al., 2005; van Haare et al., 2015; Guo et al., 2020). Although fatty acids (FAs) are the primary energy source of the heart, prolonged HFD consumption can result in lipotoxicity and increased oxidative damage of the heart. Excess lipids can also cause mitochondrial dysfunction in the heart, including decreased mitochondrial DNA copy numbers, reduced complex activities, decreased respiration, and abnormal morphology (Chen et al., 2018; Tong et al., 2019).

Mitochondrial dysfunction contributes significantly to the progression of cardiomyopathy under nutrient overload (Boudina et al., 2007; Lopaschuk et al., 2007). Mitochondrial dynamics, such as fission and fusion, is an important mitochondrial quality control process that protects mitochondria from cellular damages by remodeling mitochondrial structure and morphology (Eisner et al., 2018). Mitochondrial fission is mainly regulated by dynamin-related protein 1 (DNM1L/DRP1), while mitochondrial fusion is mediated by outer membrane proteins mitofusin 1 (MFN1) and mitofusin 2 (MFN2) or by inner membrane optic atrophy protein 1 (OPA1) (Westermann, 2012). Previous studies have demonstrated an important role of mitochondria dynamic in the cellular adaptation to various metabolic and environmental stresses (Westermann, 2012; Youle and van der bliek, 2012). Recently, mitochondrial dynamics has been linked to cardiac dysfunction. Mice with cardiac-specific *Drp1* knockout develop left ventricular dysfunction, elongated mitochondria, and reduced mitophagy (Kageyama et al., 2014; Ikeda et al., 2015). In contrast, mice cardiomyocytes with *Mfn1* knockout are protected from oxidative stress, and double knockout of *Mfn1* and *Mfn2* preserves mice hearts from acute myocardial infarction (Papanicolaou et al., 2012; Hall et al., 2016). The HFD treatment has been reported to induce mitochondria fission in both mammals (Hu et al., 2020) and *Drosophila* (Chen et al., 2020). HFD-induced mitochondrial fission depends on the activation of Drp1 in rats (Filippi et al., 2017). Thus, Drp1-dependent mitochondrial fission is an important adaptive mechanism protecting hearts from HFD-induced pathology.

The mechanistic target of rapamycin (mTOR) pathway is a highly conserved nutrient-sensing pathway that functions through two structurally and functionally distinct complexes, mTOR complex 1 (mTORC1) and mTOR complex 2 (mTORC2) (Laplante and Sabatini, 2012; Saxton and Sabatini, 2017). mTOR participates in a wide range of cellular functions, including protein synthesis, ribosomal and mitochondrial biogenesis, autophagy, and metabolism (Saxton and Sabatini, 2017). Recent studies show that mTOR signaling plays a central role in HFD-induced heart dysfunction (Birse et al., 2010). Reducing insulin-mTOR pathway prevents HFD-induced triglyceride levels and cardiac abnormalities (Birse et al., 2010; Diop et al., 2015). Additionally, increasing AMPK/mTOR activity has been observed in rats fed with HFD, which is associated with vascular dysfunction and remodeling (Ma et al., 2010). In contrast to mTORC1, mTORC2 is primarily involved in cell proliferation, cytoskeleton reorganization, lipid metabolism, stress responses, and longevity (Saltiel and Kahn, 2001; Wullschleger et al., 2006; Cybulski et al., 2009; Oh and Jacinto, 2011; Lamming et al., 2012; Lamming and Sabatini, 2013; Lamming et al., 2014).

MTORC2 also plays an important role in cardiac protection. Cardiac-specific knockout or knockdown of *Rictor*, the key subunit of mTORC2, leads to disrupted cytoskeleton organization, cardiac dilation, decreased fraction shortening, increased fibrosis, and cell death upon pressure overload and ischemic damage (Barilli et al., 2008; Volkers et al., 2013; Sciarretta et al., 2015; Shende et al., 2016). We recently show the activation of mTORC2 through overexpressing *rictor* in *Drosophila* induces autophagic activities, preserves cardiac function with aging, and prolongs lifespan (Chang et al., 2020). MTORC2 is found to localize to the mitochondrial-associated endoplasmic reticulum (ER)-membranes (MAMs) to regulate mitochondria-ER contact site, calcium influx, and mitochondrial membrane potential, suggesting a key role of mTORC2 in mitochondrial metabolism and physiology (Betz et al., 2013). Interestingly, HFD treatment induces the phosphorylation of Akt, the downstream target of mTORC2 (Diop et al., 2015). The induction of Akt phosphorylation has been previously linked to the activation of mitochondrial fission (Kim et al., 2016). However, it remains unclear whether mTORC2 can protect hearts from HFD treatment by activating mitochondrial fission.

Here, we use the *Drosophila* model to investigate the role of mTORC2 in HFD-induced mitochondrial fission and cardiac dysfunction. *Drosophila* heart shares similarities with the vertebrate heart regarding the heart development during embryogenesis and cardiomyocyte senescence (Bodmer, 1995; Harvey, 1996; Blice-Baum et al., 2019). In addition, *Drosophila* has recently emerged as an important model for investigating the genetic mechanisms underlying HFD-induced obesity and cardiac dysfunction (Birse et al., 2010; Diop et al., 2015; Guida et al., 2019). In this study, we show that HFD feeding induces mitochondrial fragmentation and cardiac contractile dysfunction. Interestingly, cardiac-specific knockdown of *rictor* blocks HFD-induced mitochondrial fragmentation and Drp1 recruitment, and it also exaggerates contractile defects. Surprisingly, cardiac-specific knockdown of Akt, the major target of mTORC2, did not block HFD-induced mitochondrial fragmentation. Lastly, knockdown of *Drp1* phenocopies *rictor* knockdown in blocking HFD-induced fragmentation and inducing contractile defects. Thus, our findings reveal that mTORC2 protects fly hearts from HFD treatment through Drp1-dependent mitochondrial fission.

## Materials and methods

### Fly stocks

Flies were maintained at 25°C, 60% relative humidity and 12 h light/dark cycles. Female flies (∼1 week of age) were reared on the standard cornmeal and yeast-based diet (0.8% cornmeal, 10% sugar, and 2.5% yeast) or the HFD (standard cornmeal and yeast-based diet supplemented with 20% w/v coconut oil). To induce gene expression in the GeneSwitch lines, RU food was made by adding 100 ul of 200 μM RU486 (Mifepristone, Cayman Chemical #100063171) onto the surface of fly food. Fly stocks used in this study were as follows: *Hand4.2-Gal4* (a gift from Rolf Bodmer), *TinCΔ4-Gal4* (a gift from Rolf Bodmer), *Hand. myo-Gal4* (a gift from Hui-Ying Lim), *Hand-GS-Gal4* (a gift from Rolf Bodmer), *da-GS-Gal4* (a gift from Véronique Monnier), *UAS-rictor RNAi* (#1-BDSC #31527, #2-BDSC #36584), *rictor* ^*Δ24*^ (a gift from Jongkyeong Chung), *UAS-rictor-OE* (a gift from Ernst Hafen), *UAS-Akt RNAi* (BDCS #82957), *UAS-Drp1 RNAi* (#1-BDSC #51483, #2- BDSC #27682), *UAS-Drp1-OE* (BDSC #51647), *Drp1* ^*[CR00300]*^ (BDCS #79236). *yw* ^*R*^ flies (a gift from Eric Rulifson) were used as control or wild-type flies.

### Western blot analysis of *Drosophila* heart

About 20 fly hearts per condition were lysed with 20 mM Tris-HCl (pH 8.0), 100 mM NaCl buffer supplied with protease inhibitor at 4°C for 20 min. After centrifugation at 14,000 rpm for 30 min, the supernatants were denatured with Laemmli sample buffer at 95°C for 5 min. Proteins were separated by Mini-PROTEAN^®^ TGX Precast Gels (Bio-Rad Laboratories, Inc.), transferred to PVDF membranes, immunoblotted with the following primary and secondary antibodies, and visualized with Pierce ECL Western Blotting Substrate. The antibodies used in western blot were: anti-phospho-Akt (Ser473) (Cell signaling technology #4060, 1:1000), anti-Akt (pan) (Cell signaling technology #4691, 1:2000), anti-beta-actin (Cell signaling technology #4967, 1:2000), Peroxidase AffiniPure Donkey Anti-Rabbit IgG (Jackson ImmunoResearch # 711-035-152, 1:5000).

### Immunofluorescent staining of *Drosophila* heart

Each fly heart sample was dissected in Schneider’s *Drosophila* medium (ThermoFisher #217-20024) and fixed with 4% paraformaldehyde (Electron Microscopy Sciences #15710) for 20 min at room temperature. Then, the samples were washed with 1x Phosphate Buffered Saline with 0.3% Triton X-100 (Fisher scientific #151-500) (0.3% PBST). After blocking the samples with 5% normal donkey serum (NDS, Jackson ImmunoResearch #017-000-121) for 1 h under room temperature (diluted in 0.3% PBST), the samples were stained with primary antibody diluted in 5% NDS for 16–24 h at 4°C. On the next day, after washing with 0.3% PBST, the samples were incubated in secondary antibody for 1–2 h at room temperature. The samples were then washed and mounted on the slides using ProLong Diamond Antifade Mountant (Thermo Fisher #P36961). The samples were imaged with an FV3000 Confocal Laser Scanning Microscope (Olympus). The following antibodies were used in immunostaining: anti-phospho-Akt (Ser473) (Cell signaling technology #4060, 1:200), anti-ATP5A1 for mitochondrial staining (Thermo Fisher #439800, 1:200), anti-*Drosophila* Drp1 (a gift from Leo Pallanck, 1:200), anti-mouse IgG-Alexa Fluor^®^ 488 (Jackson ImmunoResearch #715-545-150, 1:400); anti-rabbit IgG-Alexa Fluor^®^ 594 (Jackson ImmunoResearch #711-585-152, 1:400). DAPI (Fisher scientific #NC1526409) or Hoechst 33342 (Thermo Fisher #H3570) was used for nuclear staining. F-actin was stained with Phalloidin-CF^®^647 (Biotium #00041T, 1:40).

### Imaging analysis and quantification

The images were processed and analyzed by using Olympus cellSens Dimensions software and ImageJ/Fiji. For phospho-Akt quantification, the area around the nucleus of cardiomyocytes was selected as the region of interest (ROI), the mean intensity of the fluorescence was quantified using the count and measure function in cellSens Dimensions software. For mitochondrial diameter measurement, the A2 or A3 segment of the heart was selected as the ROI. Then the fluorescent threshold was adjusted to highlight mitochondria, followed by mitochondrial size correction using the object split tool in cellSens Dimensions. The maximum diameter of each mitochondrion in the ROI was quantified using the count and measure function. The maximum diameter is defined as the longest distance between two boundary points of the object. The proportion of elongated mitochondria with a maximum diameter greater than 2 μm was presented in each figure. See Supplementary Figure S1 for the workflow of mitochondrial diameter quantification**.** The colocalization between Drp1 and mitochondrial marker ATP5A1 in each ROI was measured by using the colocalization function of the cellSens and presented as Pearson correlation coefficient values.

### Complex I and IV activity assay

Mitochondrial complex I and IV activities were measured according to a previous method (Spinazzi et al., 2012). To isolate mitochondria, about 20 flies were first homogenized in a glass dunce grinder containing sucrose homogenization buffer (20 mM Tris-HCl, 40 mM KCl, 2 mM EDTA). The fly homogenate was cleared by centrifugation at 600 x g for 10 min at 4°C twice. The supernatant was transferred to a new centrifuge tube each time. After centrifugation at 14,000 × g for 10–20 min, the mitochondrial-enriched fraction in the pellet was resuspended with 10 mM Tris-HCl. Mitochondrial extracts were frozen–thawed for three times prior to the complex I and IV activity assay. For complex I activity assay, about 300 μL of complex I assay buffer (50 mM of potassium phosphate buffer pH 7.5, 0.5 mg/ml of bovine serum albumin, 0.3 mM of potassium cyanide, 0.1 mM of NADH) and 4 μg of mitochondrial extracts were mixed in 96-well plates for each sample. Six μl of 10 mM ubiquinone (Sigma #C7956) was added to each sample to initiate the reaction. Complex I inhibitor rotenone was added separately to calculate complex I specific activities. The absorbance at 340 nm was recorded for 20 min using an Agilent BioTek Gen5 plate reader. In complex IV activity assay, 200 μL Complex IV activity buffer (80ul ddH2O, 100ul 0.1 M ph7.0 potassium phosphate buffer, and 150 μL 0.5 mM reduced cytochrome C) and 0.5 μg of mitochondrial extracts were mixed in 96-well plates for each sample. Complex IV inhibitor potassium cyanide was used to obtain complex IV specific activities. The absorbance at 550 nm was recorded for 5 min using an Agilent BioTek Gen5 plate reader. The complex I and IV activities were calculated by subtracting the activity in the presence of inhibitors from the total activity following the below equation:

Complex I and IV activity (μmol min^−1^ mg^−1^) = (Δ Absorbance/min × 1,000)/[(extinction coefficient × volume of sample used in ml) × (sample protein concentration in mg ml^−1^)].

### Heart beat analysis

The fly hearts were prepared according to previously described semi-automatic optical heartbeat analysis (SOHA) (Fink et al., 2009). The high-speed heart contraction movies at the rate of 100 frames per second were captured using the Hamamatsu ORCA-Flash4.0 digital CMOS camera (Hamamatsu Photonics) and the Olympus BX51WI microscope with a 10X water immersion lens. The cardiac contractile defects, including the non-contractility and partial conduction block, were analyzed according to a previous study (Ocorr et al., 2009; Birse et al., 2010). For a non-contractile heart, the heart shows weak or no contractile. The heart with partial conduction block beats at a different rate in different regions of the heart. Fractional shortening was calculated as (diastolic diameters—systolic diameters)/diastolic diameters.

### Statistical analysis

GraphPad Prism was used for statistical analysis. Unpaired two-tailed Student’s t test was performed to compare the mean value between control and treatment groups. Two-way ANOVA followed by Tukey’s multiple comparison test was used to examine the effects of diet and gene knockdown on mitochondrial diameter/function and cardiac function. Chi-square test was performed to examine the relationship between diet (or genotype) and contractile defects. The outliers were excluded using robust regression and outlier removal method (Q = 10%) prior to the data analysis.

## Results

### mTORC2 regulates HFD-induced mitochondrial fission in *Drosophila* hearts

**FIGURE 1:**
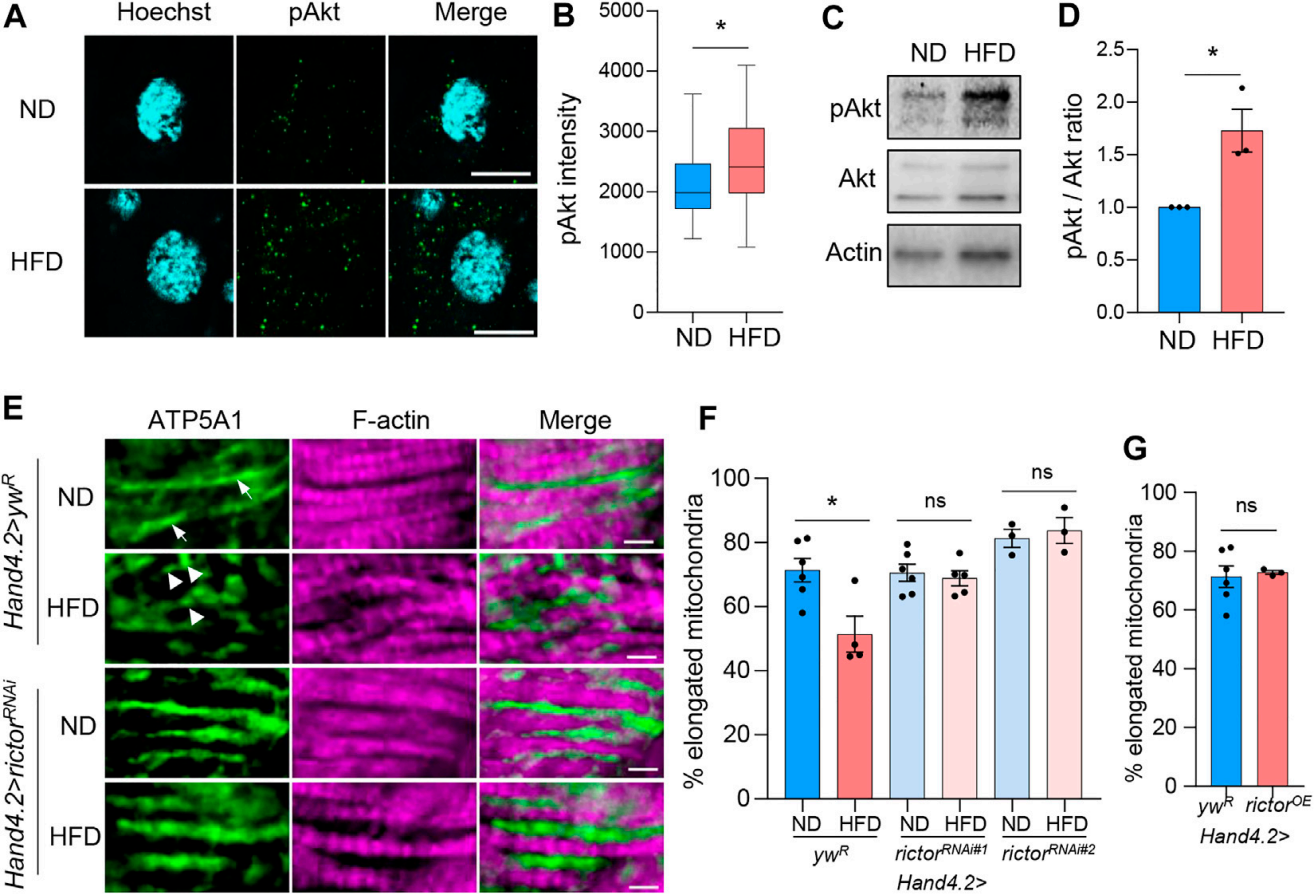
mTORC2 regulates HFD-induced mitochondrial fission in *Drosophila* hearts. **(A)** Immunostaining of phospho-Akt in fly cardiomyocytes after 2 days of ND or HFD treatment. Scale bar: 10 μm. **(B)** The quantification of mean fluorescence intensity of the phospho-Akt. Student’s t-test (*p < 0.05). N = 10 (10 hearts per diet, 2 cardiomyocytes per heart). **(C)** Western blot analysis of the pAkt, Akt and beta-actin in fly hearts. **(D)** The quantification of band intensity of the phospho-Akt normalized to total Akt. Student’s t-test (*p < 0.05). N = 3 (3 replicates per diet, 20 hearts per replicate). **(E)** Immunostaining of mitochondria and F-actin of cardiomyocytes from ND- or HFD-treated control (*yw* ^*R*^) and *rictor* knockdown flies. The cardiac driver *Hand4.2-Gal4* is used to drive heart-specific knockdown. Mitochondria are visualized using an anti-ATP5A1 antibody. Scale bar: 10 μm. Arrows: elongated mitochondria. Arrowheads: fragmented mitochondria. **(F)** The proportion of the elongated mitochondria with a maximum diameter greater than 2 μm in ND- or HFD-treated control and *rictor* knockdown flies. Two-way ANOVA: Interaction between diet and genotype is significant, *p* = 0.0155. Tukey’s multiple comparison test: *p < 0.05, ns: not significant. *N* = 3∼6 (3∼6 hearts per genotype). **(G)** The proportion of the elongated mitochondria in hearts with *rictor* overexpression. Student’s t-test (ns: not significant). *N* = 3∼6 (3∼6 hearts per genotype).

To investigate the role of mTORC2 in HFD-induced mitochondrial fission in *Drosophila* hearts, we first examined how HFD treatment regulates mTORC2 activity. Wild-type flies were fed on 20% (w/v) coconut oil for 2 days before the analysis of Akt phosphorylation, a commonly used marker for mTORC2 activation metabolism (Saxton and Sabatini, 2017). Similar to a previous study (Diop et al., 2015), we found that short-term HFD treatment (2 days) significantly induced Akt phosphorylation in cardiomyocytes compared with normal diet (ND) treatment *via* immunostaining analysis (Figures 1A,B). This finding was further verified by western blot analysis on dissected fly hearts (Figures 1C,D). Together, these results suggest that HFD treatment induces mTORC2 activity in fly hearts.

Consistent with the previous studies (Filippi et al., 2017; Chen et al., 2018; Tong et al., 2019), we found that HFD treatment induced mitochondrial fragmentation in wild-type fly hearts (Figures 1E,F). Mitochondrial fragmentation was determined by counting the proportion of elongated mitochondria with a maximum diameter greater than 2 μm in a selected ROI. To determine the role of mTORC2 in HFD-induced mitochondrial fragmentation, we monitored mitochondrial morphology in fly hearts with the knockdown of *rictor*, the key subunit of mTORC2 (Figures 1E,F). *Hand4.2-Gal4* was used to drive heart-specific gene knockdown. Two different *rictor* RNAi lines were used to eliminate genetic background effects. We found that both *rictor* knockdown lines showed no HFD-induced mitochondrial fragmentation (Figures 1E,F). It is known that *Hand4.2-Gal4* drives expression in both cardiomyocytes and pericardial cells. To determine the cardiac autonomous role of rictor, we performed *rictor* knockdown using two cardiomyocyte-specific drivers, *Hand. myo-Gal4* (Liu et al., 2019) and *TinCΔ4-gal4* (Chang et al., 2020). As shown in Supplementary Figures S2A,B, cardiomyocyte-specific knockdown of *rictor* also blocked HFD-induced mitochondrial fragmentation, suggesting rictor regulates mitochondrial fission in a cardiomyocyte-dependent manner. Furthermore, to avoid the potential effects of constitutive gene knockdown on heart development, we examined adult-onset knockdown of *rictor* using a GeneSwitch heart driver, *Hand-GS-Gal4*. We found that 5∼7 days of *rictor* knockdown in adult flies significantly blocked HFD-induced mitochondrial fragmentation (Supplementary Figure S2C). Lastly, since HFD increases mTORC2 activity, we wonder if overexpressing *rictor* alone is sufficient to induce mitochondrial fission. As shown in Figure 1G, overexpression of *rictor* did not alter mitochondrial size and morphology. Taken together, these results suggest that mTORC2 is required for HFD-induced mitochondrial fission.

### Rictor is required for cardiac protection and mitochondrial function under HFD

**FIGURE 2:**
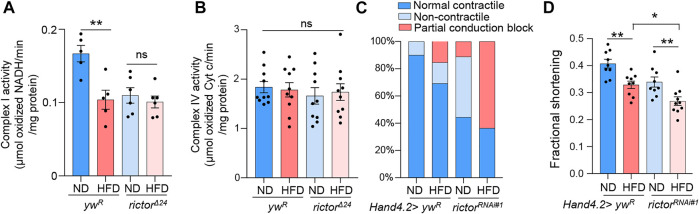
Rictor is required for cardiac protection and mitochondrial function under HFD. **(A)** The complex I activity of the wild-type and *rictor* mutant flies under ND or HFD treatment. Two-way ANOVA: Interaction between diet and genotype is significant, *p* = 0.0211. Tukey’s multiple comparison test: **p < 0.01, ns: not significant. N = 5∼6 (5∼6 replicates, 20 flies per replicate). **(B)** The complex IV activity of the wild-type and *rictor* mutant flies under ND or HFD treatment. Two-way ANOVA: Interaction between diet and genotype is not significant, *p* = 0.6614. Tukey’s multiple comparison test: ns: not significant. N = 9∼10 (9∼10 replicates, 20 flies per replicate). **(C)** The incidence of contractile defects (non-contractile and partial conduction block) of control and *rictor* knockdown flies upon HFD treatment. Chi-square test: no effects of diet on contractile defects, *X* ^2^ (1, *N* = 43) = 2.633, *p* > 0.1; Knockdown of rictor likely increases the incidence of contractile defects (especially partial conduction block) upon HFD treatment, *X* ^*2*^ (1, N = 43) = 5.135, *p* < 0.01. 15∼20 flies per condition. **(D)** The quantification of the fractional shortening of control and *rictor* knockdown flies. Two-way ANOVA: Interaction between diet and genotype is not significant, *p* = 0.8233. Tukey’s multiple comparison test: *p < 0.05, **p < 0.01. N = 9∼10 (9∼10 flies per condition).

Given the vital role of rictor in HFD-induced mitochondrial fission in fly hearts, we next asked whether rictor is involved in HFD-induced mitochondrial and cardiac dysfunction. To monitor mitochondrial function, we first measured the complex I activity. Complex I is the key component of the electron transport chain that is responsible for ATP production. We found that the complex I activity was reduced upon short-term HFD treatment (2 days), which might be due to reduced complex I abundance in fragmented mitochondria upon HFD treatment (Figure 2A). Interestingly, HFD treatment did not down-regulate complex I activity in *rictor* loss-of-function mutants, even though the complex I activity of *rictor* mutants was lower than wild-type flies before HFD treatment (Figure 2A). In addition, we measured complex IV activity to assess other electron transport processes and mitochondria function. We found that the complex IV activity was not affected by either HFD treatment or *rictor* mutants (Figure 2B). These results suggest that not all electron transport processes are altered by short-term HFD treatment, and mTORC2 specifically targets the complex I activity potentially through the regulation of mitochondrial fission upon HFD treatment.

Next, we carried out semi-automatic optical heartbeat analysis (SOHA) to determine whether mTORC2 is required for cardiac protection under HFD treatment. We fed control flies and *rictor* knockdown flies on either ND or HFD for 7 days prior to the SOHA analysis, as there are no obvious cardiac defects found upon short-term (2 days) HFD treatment (Birse et al., 2010). The incidence of abnormal contractility (e.g., partial conduction block and non-contractility) and altered fractional shortening were monitored accordingly to a previous study (Birse et al., 2010). In wild-type flies, HFD treatment slightly (but not significantly) increased the incidence of abnormal contractility (*X* ^2^ (1, *N* = 43) = 2.633, *p* > 0.1)) (Figure 2C). In contrast, knockdown of *rictor* significantly increased the incidence of contractile defects (especially partial conduction block) upon HFD treatment (*X* ^*2*^ (1, N = 43) = 5.135, *p* < 0.01)) (Figure 2C). Cardiac-specific knockdown of *rictor* also significantly decreased fractional shortening upon HFD treatment, a common measurement for heart muscular contractility (Figure 2D). Taken together, these results suggest that mTORC2 is required for cardiac protection upon HFD treatment, which might be due to its positive role in mitochondrial fission.

### Akt is not responsible for the mitochondrial fission under HFD treatment

**FIGURE 3:**
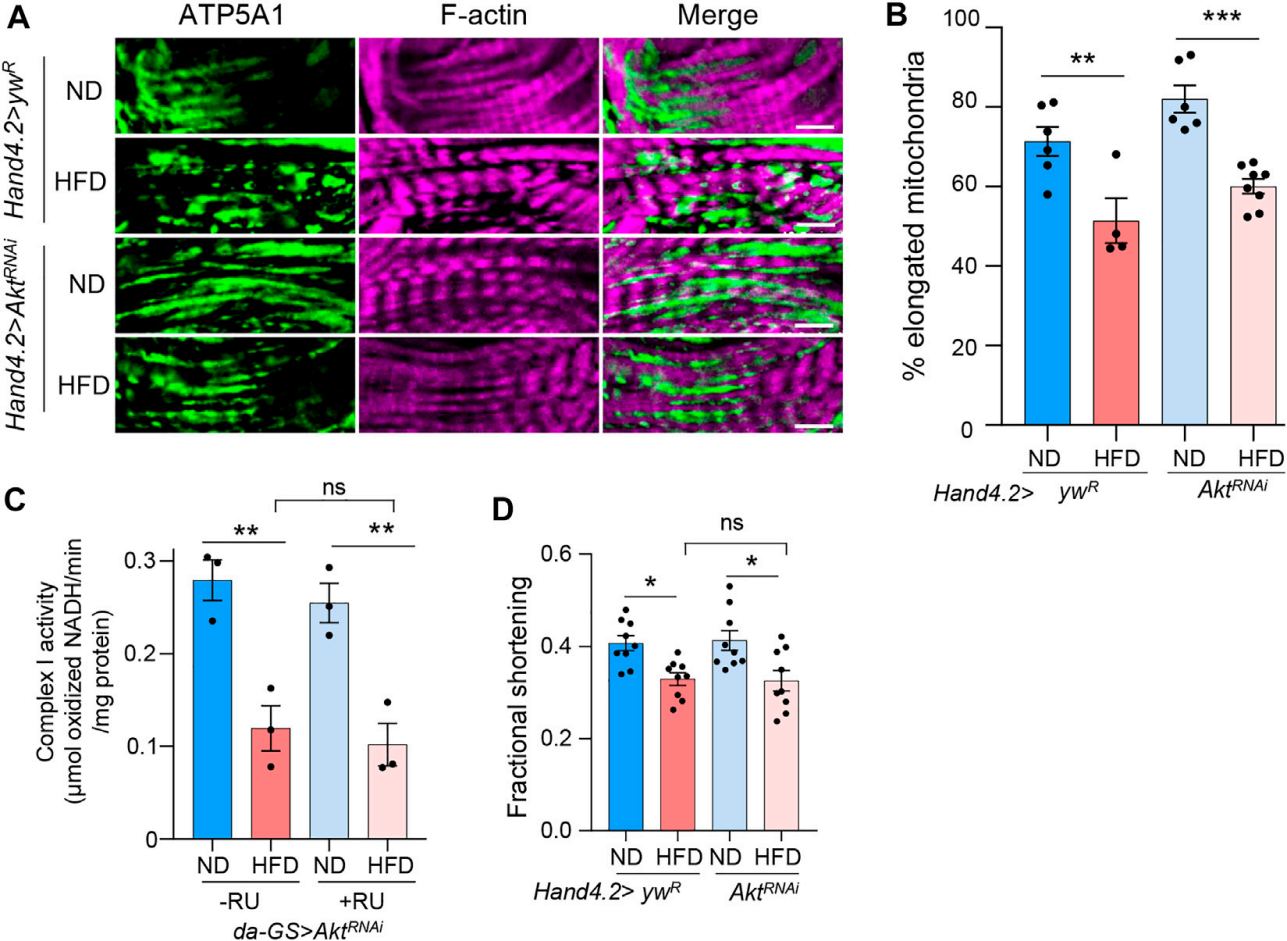
Akt is not responsible for mitochondrial fission under HFD treatment. **(A)** Immunostaining of mitochondria and F-actin of cardiomyocytes from ND- or HFD-treated control and *Akt* knockdown flies. Mitochondria are visualized using an anti-ATP5A1 antibody. Scale bar: 10 μm. **(B)** The proportion of the elongated mitochondria of the control and *Akt* knockdown flies under ND or HFD treatment. Two-way ANOVA: Interaction between diet and genotype is not significant, *p* = 0.7747. Tukey’s multiple comparison test: **p < 0.01, ***p < 0.001. N = 6∼8 (6∼8 hearts per genotype). The control group re-uses the data from Figure 1F. **(C)** The complex I activity of the control and *Akt* knockdown flies under ND or HFD treatment. Two-way ANOVA: Interaction between diet and genotype is not significant, *p* = 0.88. Tukey’s multiple comparison test: **p < 0.01, ns: not significant. *N* = 3 (3 replicates, 20 flies per replicate). **(D)** The quantification of the fractional shortening of control and *Akt* knockdown flies. Two-way ANOVA: Interaction between diet and genotype is not significant, *p* = 0.2564. Tukey’s multiple comparison test: *p < 0.05, ns: not significant. N = 9∼10 (9∼10 flies per condition).

Next, we investigated the mechanisms for how mTORC2 controls mitochondrial fission under HFD treatment. Given that Akt phosphorylation is induced under HFD treatment, we decided to test whether mTORC2 regulates mitochondrial fission through its downstream effector Akt. We knocked down *Akt* in fly hearts using *Hand4.2-Gal4* driver and monitored mitochondrial fragmentation upon HFD treatment. Surprisingly, we found that knockdown of *Akt* did not block HFD-induced mitochondrial fragmentation (Figures 3A,B), which suggests that Akt is not responsible for HFD-induced mitochondrial fission. In addition, we examined whether *Akt* knockdown affects mitochondrial and cardiac function. Unlike *rictor* knockdown, we found that *Akt* knockdown did not alter the down-regulation of complex I activity and fractional shortening by HFD treatment (Figures 3C,D). Thus, mTORC2 may control mitochondrial fission through a different downstream target.

### Rictor controls mitochondrial fission through Drp1 under HFD treatment

**FIGURE 4:**
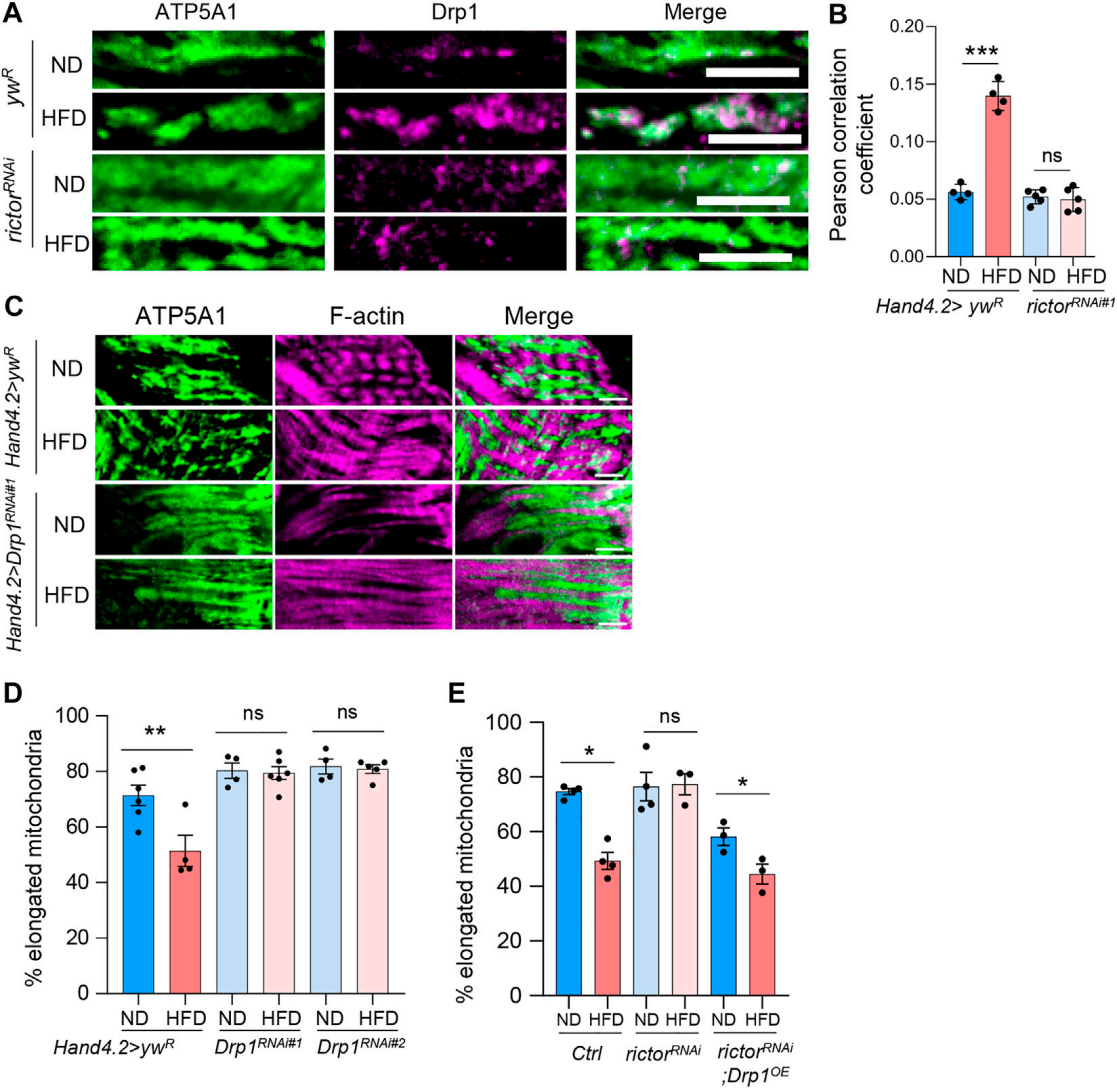
Rictor controls HFD-induced mitochondrial localization of Drp1. **(A)** Immunostaining of mitochondrial localization of Drp1 protein in cardiomyocytes of ND- or HFD-treated control and *rictor* knockdown flies. Scale bar: 10 μm. **(B)** The quantification of Pearson correlation coefficient of the colocalization between Drp1 and mitochondrial marker (ATP5A1). Two-way ANOVA: Interaction between diet and genotype is highly significant, *p* < 0.0001. Tukey’s multiple comparison test: ***p < 0.001, ns: not significant. N = 4∼5 (4∼5 hearts per genotype). **(C)** Immunostaining of mitochondria and F-actin of cardiomyocytes from ND- or HFD-treated control and *Drp1* knockdown flies. Scale bar: 10 μm. **(D)** The proportion of the elongated mitochondria of the control and *Drp1* knockdown flies under ND or HFD treatment. Two-way ANOVA: Interaction between diet and genotype is significant, *p* = 0.0098. Tukey’s multiple comparison test: **p < 0.01, ns: not significant. N = 4∼6 (4∼6 hearts per genotype). The control group re-uses the data from Figure 1F. **(E)** The proportion of the elongated mitochondria of the control, *rictor* knockdown, and *rictor* ^*RNAi*^ *; Drp1* ^*OE*^ flies under ND or HFD treatment. Two-way ANOVA: Interaction between diet and genotype is significant, *p* = 0.0072. Tukey’s multiple comparison test: *p < 0.05, ns: not significant. N = 3∼4 (3∼4 hearts per genotype).

To explore the factors involved in rictor-regulated mitochondrial fission upon HFD treatment, we examined the fission protein, Drp1. The correlation between mTORC2 activity and Drp1 activation has been previously reported (Kim et al., 2016). Since the recruitment of Drp1 to the mitochondria is one of the essential steps of mitochondrial fission, we co-stained Drp1 and a mitochondrial marker (ATP synthase subunit ATP5A1) to monitor the Drp1 recruitment to mitochondria under HFD treatment. As expected, HFD treatment increased the colocalization of Drp1 and mitochondrial marker ATP5A1, while *rictor* knockdown blocked HFD-induced mitochondrial localization of Drp1 (Figures 4A,B).

Similar to *rictor* knockdown, we found that knockdown of *Drp1* in fly hearts blocked HFD-induced mitochondrial fragmentation (Figures 4C,D). Two *Drp1* RNAi lines were used. We also showed that Drp1-mediated regulation of mitochondrial fission is cardiac autonomous by knocking down *Drp1* using two cardiomyocyte-specific drivers, *Hand. myo-Gal4* and *TinCΔ4-gal4* (Supplementary Figures S2D,E). Furthermore, adult-onset knockdown of *Drp1* also blocked HFD-induced mitochondrial fragmentation (Supplementary Figure S2F). To determine whether Drp1 acts as the downstream effector of mTORC2 to regulate HFD-induced mitochondrial fission, we conducted an epistasis analysis using a combined fly line, *Hand-GS-Gal4; rictor RNAi*, and crossed it with control or *Drp1* overexpression flies. As shown in Figure 4E, knockdown of *rictor* alone blocked HFD-induced mitochondrial fragmentation, while overexpression of *Drp1* restored HFD-induced mitochondrial fragmentation in *rictor* knockdown flies. Together, our findings suggest that mTORC2 regulates HFD-induced mitochondrial fission through Drp1 recruitment to mitochondria.

**FIGURE 5:**
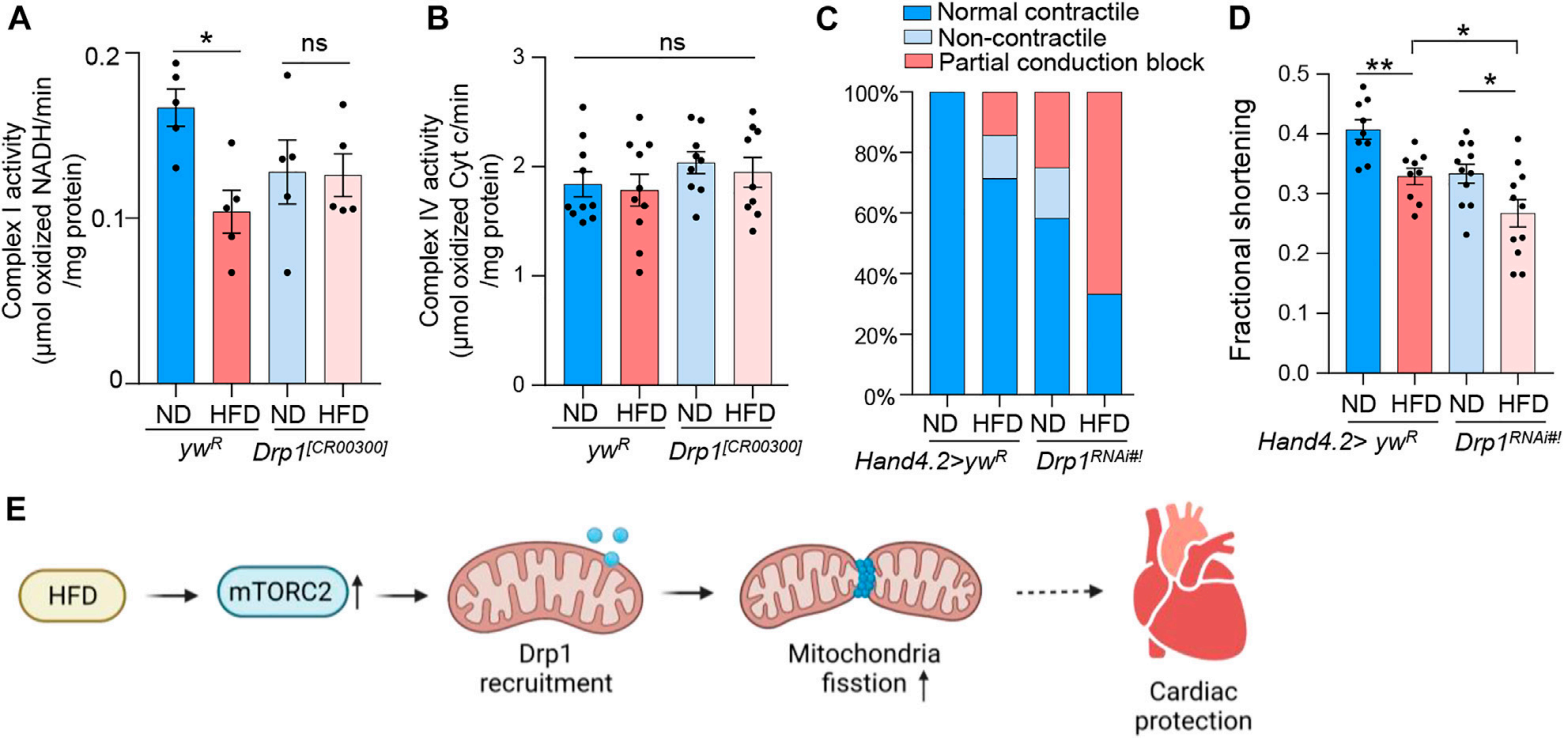
Drp1 regulates mitochondrial and cardiac dysfunction under HFD treatment. **(A)** The complex I activity of the wild-type and *Drp1* mutant flies under ND or HFD treatment. Two-way ANOVA: Interaction between diet and genotype is significant, *p* = 0.05. Tukey’s multiple comparison test: *p < 0.05, ns: not significant. N = 5∼6 (5∼6 replicates, 20 flies per replicate). The control group re-uses the data from Figure 2A. **(B)** The complex IV activity of the wild-type and *Drp1* mutant flies under ND or HFD treatment. Two-way ANOVA: Interaction between diet and genotype is not significant, *p* = 0.8954. Tukey’s multiple comparison test: ns: not significant. *N* = 9∼10 (9∼10 replicates, 20 flies per replicate). The control group re-uses the date from Figure 2B. **(C)** The incidence of contractile defects (non-contractile and partial conduction block) of control and *Drp1* knockdown flies upon HFD treatment. Chi-square test: Knockdown of *Drp1* likely increases the incidence of contractile defects (especially partial conduction block) upon HFD treatment, *X* ^2^ (1, *N* = 50) = 7.837, *p <* 0.01. 15∼20 flies per condition. The control group re-uses the data from Figure 2C. **(D)** The quantification of the fractional shortening of control and *Drp1* knockdown flies. Two-way ANOVA: Interaction between diet and genotype is not significant, *p* = 0.7507. Tukey’s multiple comparison test: *p < 0.05, **p < 0.01. N = 9∼11 (9∼11 flies per condition). The control group re-uses the data from Figure 2D. **(E)** The working model showing a novel role of mTORC2 in regulating HFD-induced mitochondrial fission and cardiac dysfunction (created with BioRender.com).

To further examine the role of Drp1 in the regulation of HFD-induced mitochondrial and cardiac dysfunction, we measured complex I and IV activities, contractile defects, and fractional shortening of the *Drp1* knockdown and mutant flies. Similar to *rictor* mutants, HFD treatment did not reduce complex I activity of *Drp1* loss-of-function (Figure 5A), while the complex IV activity was not affected by both HFD treatment and *Drp1* mutants (Figure 5B). Furthermore, heart-specific knockdown of *Drp1* exaggerated the detrimental effects of HFD on cardiac contractile function (*X* ^2^ (1, *N* = 50) = 7.837, *p <* 0.01) (Figure 5C) and fractional shortening defects (Figure 5D). Taken together, these results suggest that Drp1 might be an important downstream effector of mTORC2 in protecting hearts under HFD treatment.

## Discussion

In this study, we uncover a novel role of mTORC2 in regulating HFD-induced mitochondrial fission and cardiac dysfunction (Figure 5E). We show that mTORC2 activity is activated by short-term HFD treatment, while the repression of mTORC2 through cardiac-specific *rictor* knockdown blocks mitochondrial fission and exaggerates cardiac defects under HFD feeding. We further show that mTORC2 regulates mitochondrial fission through Drp1 recruitment to mitochondria. Deletion of either *Rictor* or *Drp1* is detrimental to mouse hearts (Barilli et al., 2008; Volkers et al., 2013; Kageyama et al., 2014; Ikeda et al., 2015; Sciarretta et al., 2015; Shende et al., 2016). Thus, our findings suggest that the activation of mTORC2 under HFD treatment is an important adaptive mechanism to protect hearts through Drp1-dependent mitochondrial fission.

The mTORC2 pathway plays important roles in cell survival, cytoskeleton reorganization, metabolism, and lifespan (Saltiel and Kahn, 2001; Wullschleger et al., 2006; Cybulski et al., 2009; Oh and Jacinto, 2011; Lamming et al., 2012; Lamming and Sabatini, 2013; Lamming et al., 2014). However, our knowledge of mTORC2 and mitochondrial homeostasis is limited. Although mTORC2 has been implicated in mitochondrial quality control through the interactions with tricornered (trc) kinases and autophagy (Wu et al., 2013; Aspernig et al., 2019; Chang et al., 2020), the potential role of mTORC2 in mitochondrial dynamics remains largely unknown. Our genetic analyses uncover a vital function of mTORC2 in HFD-induced mitochondrial fission and Drp1 recruitment, suggesting a new way used by mTORC2 to maintain mitochondrial homeostasis. MTORC2 has been shown to localize to mitochondrial-associated endoplasmic reticulum (ER)-membranes (MAM), and knockout of *Rictor* disrupts MAM and mitochondrial membrane potential and calcium uptake (Betz et al., 2013). MAM is suggested as an important site for mitochondrial fission (Marchi et al., 2014), and the elevated cytosolic Ca^2+^ has been linked to mitochondrial fission in rat cardiomyocytes (Hom et al., 2010). MTORC2 might likely regulate mitochondrial fission through ER-mitochondrial contact site and calcium signaling.

AKT is one of the major downstream effectors of mTORC2 signaling in the regulation of cellular growth, survival, actin cytoskeleton (Jacinto et al., 2004; Sarbassov et al., 2005). AKT has also been linked to cardiac protective factors during ischemic damage (Volkers et al., 2013; Yano et al., 2014). Surprisingly, we found that Akt is not required for HFD-induced mitochondrial fission in fly hearts, although the phosphorylation of Akt is induced by HFD treatment. These findings suggest that mTORC2 might regulate mitochondrial fission through other downstream effectors. It is known that mTORC2 can target Rho GTPases Rac1 to control the polymerization of actin (Jacinto et al., 2004). The recruitment of Drp1 to mitochondria requires actin filament, and inhibition of actin polymerization can disrupt Drp1recruitment (Ji et al., 2015). Thus, Rac1-regulated actin cytoskeleton might be one possible mechanism for mTORC2-regulated mitochondrial fission.

HFD is known to induce oxidative stress (Matsuzawa-Nagata et al., 2008), which might be one of the causes of mTORC2 activation (Cai and Andres, 2014; Dhar et al., 2019). The induction of mitochondrial fission has been reported as an adaptive response to intracellular oxidative damage to mitochondria (Wu et al., 2011; He et al., 2018; Hu et al., 2019; Sun et al., 2020). Therefore, mTORC2-mediated mitochondrial fission is likely a cytoprotective response to elevated oxidative stress upon HFD treatment. One possible way that mTORC2 senses oxidative stress might be through mTORC2 subunit SIN1/MAPKAP1 protein, as oxidative stress can induce the binding between SIN1 and c-Jun N-terminal kinase (JNK) (Schroder et al., 2005; Wu et al., 2009). However, the detailed mechanism for how mTORC2 senses oxidative stress needs to be further determined.

In summary, we uncover a new cardiac protection mechanism by which mTORC2 promotes mitochondrial fission in responding to HFD treatment. Our findings provide new insights into mTORC2-regulated mitochondrial homeostasis in animal hearts. In addition, our studies underscore a promising therapeutic strategy to combat HFD-induced cardiomyopathy by targeting mTORC2 signaling.

## Data Availability

The original contributions presented in the study are included in the article/Supplementary material, further inquiries can be directed to the corresponding author.

## Supplementary material

Supplementary Figure S1 The workflow of mitochondrial diameter quantification. The maximum diameter is defined as the longest distance between two boundary points of the object. The proportion of elongated mitochondria with a maximum diameter greater than two μm is presented in each figure.

Supplementary Figure S2 Examine the role of rictor and Drp1 in HFD-induced mitochondrial fragmentation using two cardiomyocyte-specific drivers and one GeneSwitch driver. **(A)** The proportion of the elongated mitochondria of the control and rictor knockdown flies using cardiomyocyte-specific driver Hand.myo-Gal4. Two-way ANOVA: Interaction between diet and genotype is significant, *p* = 0.0182. **(B)** The proportion of the elongated mitochondria of the control and rictor knockdown flies using cardiomyocyte-specific driver TinCΔ4-Gal4. Two-way ANOVA: Interaction between diet and genotype is significant, *p* = 0.04. **(C)** The proportion of the elongated mitochondria of the control and rictor knockdown flies using GeneSwitch driver Hand-GS-Gal4. Two-way ANOVA: Interaction between diet and genotype is significant, *p* = 0.0013. **(D)** The proportion of the elongated mitochondria of the control and Drp1 knockdown flies using cardiomyocyte-specific driver Hand.myo-Gal4. Two-way ANOVA: Interaction between diet and genotype is significant, *p* = 0.021. **(E)** The proportion of the elongated mitochondria of the control and Drp1 knockdown flies using cardiomyocyte-specific driver TinCΔ4-Gal4. Two-way ANOVA: Interaction between diet and genotype is significant, *p* = 0.0088. **(F)** The proportion of the elongated mitochondria of the control and Drp1 knockdown flies using GeneSwitch driver Hand-GS-Gal4. Two-way ANOVA: Interaction between diet and genotype is significant, *p* = 0.0001. Tukey’s multiple comparison test: * *p* < 0.05, *** *p* < 0.001, ns: not significant. *N* = 4∼6 (4∼6 hearts per genotype).
