# Supplementary material for: mTORC2 protects the heart from high-fat diet-induced cardiomyopathy through mitochondrial fission in Drosophila
Source: Front Cell Dev Biol. 2022 Jul 15;10:866210. doi: 10.3389/fcell.2022.866210 (PMC9334792; doi:10.3389/fcell.2022.866210)
Supplement: Supplementary file 1 [file Presentation1.pdf]

Supplementary Material

mTORC2 protects the heart from high-fat diet-induced cardiomyopathy through mitochondrial fission in *Drosophila*

Peiduo Liu[1†], Kai Chang[1†], Guillermo Requejo[1], Hua Bai[1*]

[1]Department of Genetics, Development, and Cell Biology, Iowa State University, Ames, IA 50011, USA

Supplementary Figure S1

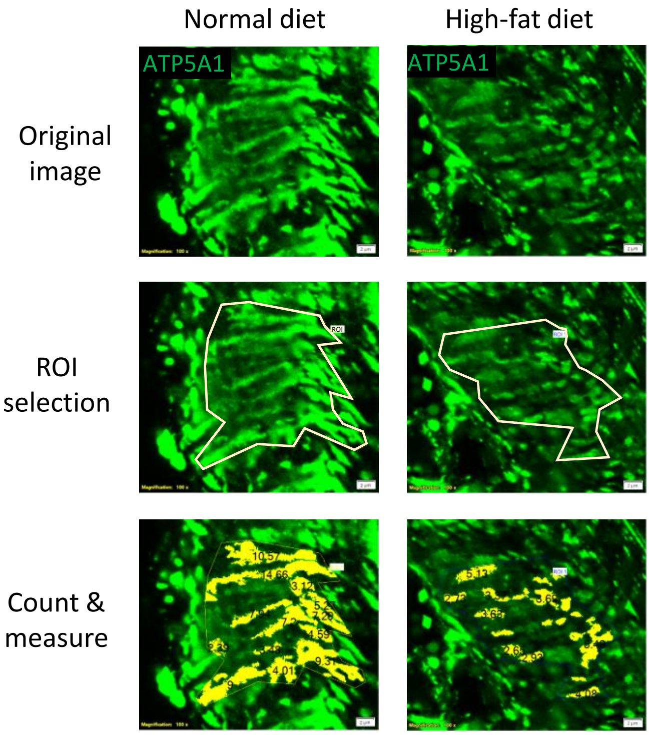

Procedures:

1. Open original image in Olympus cellSens software

2. Select cardiomyocyte region using polygon ROI selection tool

3. Adjust threshold to highlight mitochondria
4. Use object split tool for mitochondrial size correction
5. Use count and measure tool to quantify maximum diameter of selected mitochondria

Data output:

Max diameter (µm)	Obj. 1	Obj. 2	Obj. 3	Obj. 4	Obj. 5	Obj. 6	Obj. 7	Obj. 8	Obj. 9	Obj. 10	Obj. 11	Obj. 12	Obj. 13	Obj. 14	Obj. 15	Obj. 16	Obj. 17	Obj. 18	Obj. 19	% elongated mitochondria (>2 µm)
Normal diet	14.66	10.57	9.31	9	7.94	7.29	7.2	5.27	4.59	4.01	3.48	3.12	2.39	1.76	1.53	/	/	/	/	86.70%
High-fat diet	5.49	5.13	4.08	3.69	3.63	3.37	2.93	2.73	2.65	2.5	2.42	2.26	1.92	1.79	1.77	1.52	1.49	1.25	1.19	63.10%

Supplementary Figure S1. The workflow of mitochondrial diameter quantification. The maximum diameter is defined as the longest distance between two boundary points of the object. The proportion of elongated mitochondria with a maximum diameter greater than two µm is presented in each figure.

Supplementary Figure S2

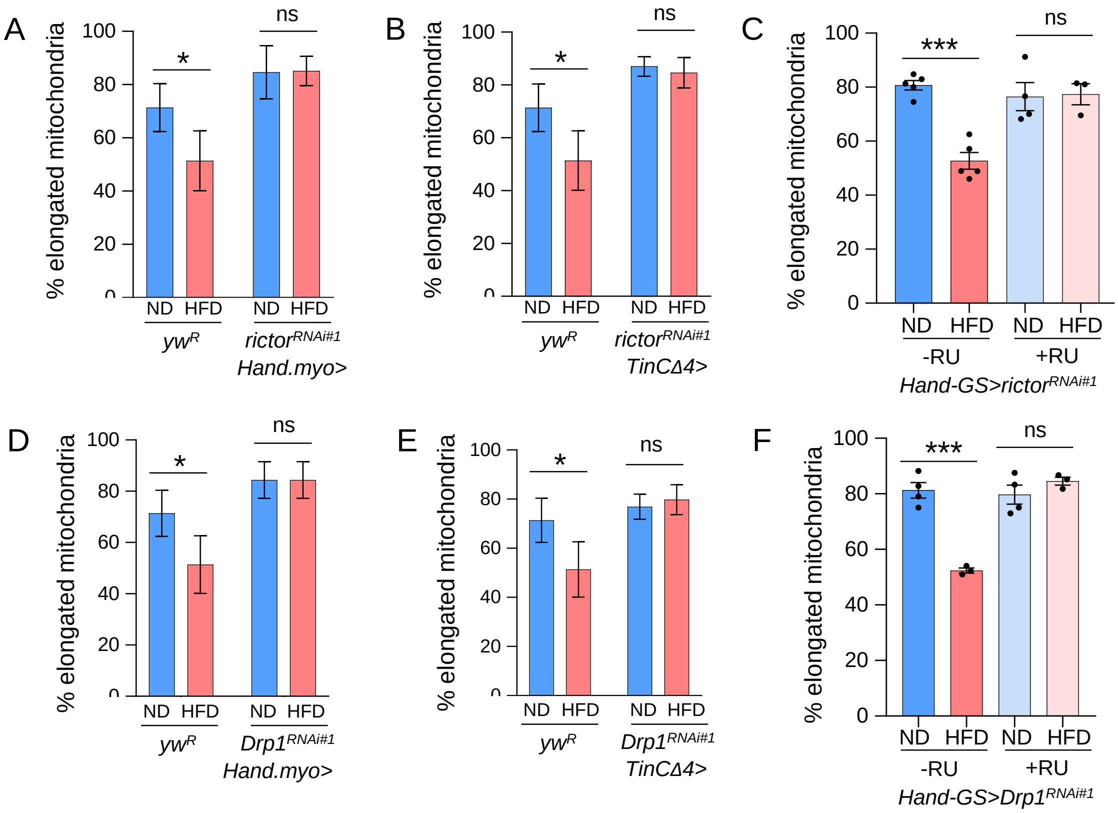

Supplementary Figure S2. Examine the role of rictor and Drp1 in HFD-induced mitochondrial fragmentation using two cardiomyocyte-specific drivers and one GeneSwitch driver. **(A)** The proportion of the elongated mitochondria of the control and *rictor* knockdown flies using cardiomyocyte-specific driver *Hand.myo-Gal4*. Two-way ANOVA: Interaction between diet and genotype is significant, p=0.0182. **(B)** The proportion of the elongated mitochondria of the control and *rictor* knockdown flies using cardiomyocyte-specific driver *TinCΔ4-Gal4*. Two-way ANOVA: Interaction between diet and genotype is significant, p=0.04. **(C)** The proportion of the elongated mitochondria of the control and *rictor* knockdown flies using GeneSwitch driver *Hand-GS-Gal4*. Two-way ANOVA: Interaction between diet and genotype is significant, p=0.0013. **(D)** The proportion of the elongated mitochondria of the control and *Drp1* knockdown flies using cardiomyocyte-specific driver *Hand.myo-Gal4*. Two-way ANOVA: Interaction between diet and genotype is significant, p=0.021. **(E)** The proportion of the elongated mitochondria of the control and *Drp1* knockdown flies using cardiomyocyte-specific driver

TinCΔ4-Gal4. Two-way ANOVA: Interaction between diet and genotype is significant, p=0.0088. **(F)** The proportion of the elongated mitochondria of the control and *Drp1* knockdown flies using GeneSwitch driver *Hand-GS-Gal4*. Two-way ANOVA: Interaction between diet and genotype is significant, p=0.0001. Tukey's multiple comparison test: * p<0.05, *** p<0.001, ns: not significant. N=4~6 (4~6 hearts per genotype).
